# Endogenous and Synthetic Cannabinoids as Therapeutics in Retinal Disease

**DOI:** 10.1155/2016/8373020

**Published:** 2016-01-06

**Authors:** Despina Kokona, Panagiota-Christina Georgiou, Mihalis Kounenidakis, Foteini Kiagiadaki, Kyriaki Thermos

**Affiliations:** ^1^University of Crete, School of Medicine, Department of Pharmacology, Heraklion, 71003 Crete, Greece; ^2^Department of Ophthalmology, Bern University Hospital, Inselspital, 3010 Bern, Switzerland

## Abstract

The functional significance of cannabinoids in ocular physiology and disease has been reported some decades ago. In the early 1970s, subjects who smoked* Cannabis sativa* developed lower intraocular pressure (IOP). This led to the isolation of phytocannabinoids from this plant and the study of their therapeutic effects in glaucoma. The main treatment of this disease to date involves the administration of drugs mediating either the decrease of aqueous humour synthesis or the increase of its outflow and thus reduces IOP. However, the reduction of IOP is not sufficient to prevent visual field loss. Retinal diseases, such as glaucoma and diabetic retinopathy, have been defined as neurodegenerative diseases and characterized by ischemia-induced excitotoxicity and loss of retinal neurons. Therefore, new therapeutic strategies must be applied in order to target retinal cell death, reduction of visual acuity, and blindness. The aim of the present review is to address the neuroprotective and therapeutic potential of cannabinoids in retinal disease.

## 1. Introduction

The study of cannabinoids in the central nervous system (CNS) has been the primary focus of many investigations, not only due to their psychotropic effects, but also due to their involvement in neuroplasticity and their therapeutic use in conditions such as obesity, pain, and neurodegenerative diseases. Fewer studies have focused on the role of cannabinoids in the retina, as well as other critical players of the visual system [1, 2].

The functional significance of the cannabinoids in ocular physiology and disease was noted by Hepler and Frank [3] who reported that subjects who smoked marijuana (*Cannabis sativa*) developed lower intraocular pressure (IOP). This finding was subsequently reproduced and the results suggested that cannabinoids isolated from cannabis may be useful in the treatment of glaucoma [4–6].

The presence of a functional endocannabinoid system in the retina, which includes (a) endogenous cannabinoids, (b) enzymes involved in their synthesis and metabolism, and (c) cannabinoid receptors, supports a role for cannabinoids in retinal circuitry and vision [1]. Arachidonoyl ethanolamide (anandamide, AEA) and 2-arachidonoylglycerol (2-AG) have been found in retinas of many species [7–10].

Two receptors have been cloned, CB1 and CB2, that mediate the physiological and pharmacological actions of endocannabinoids, as well as the actions of the natural and synthetic cannabinoids [11–15]. CB1 receptors are predominant in the CNS and are expressed in brain areas that influence motor and cognitive functions [1, 2], as well as in areas that comprise the visual pathway of the brain, namely, the thalamus and visual cortex [16–18]. CB2 receptors are also found in the CNS, but their location and function are mainly in peripheral tissues and the immune system [19, 20].

The aim of this review is to summarize the knowledge acquired to date on the function of endogenous and synthetic cannabinoids in the retina and to address the neuroprotective and therapeutic potential of cannabinoids in retinal disease.

## 2. Endocannabinoid System in Retina

### 2.1. Cannabinoid Receptor Localization in Retinal Neurons

Immunohistochemical studies described the presence and localization of the CB1 receptor in retinal neurons of several animal species [10]. Specifically, CB1 immunoreactivity was detected in the inner and outer plexiform layers (and/or on cone pedicles and rod spherules) of tiger salamander, gold fish, chick, mouse, rat, and rhesus monkey, in amacrine and retinal ganglion cells (RGCs) and ganglion cell axons of all species except gold fish, and sparsely labeled photoreceptors of monkey, mouse, rat, and chicken. CB1 immunoreactivity has been detected in rod bipolar cells, GABA-amacrine cells, and horizontal cells [21]. In the mouse retina, a specific cannabinoid circuitry has been defined based on colocalization of the CB1 receptor and a repertoire of proteins that influence the endocannabinoid system [22].

The expression of CB2 cannabinoid receptor mRNA has been reported in adult rat retina, as well as in human retinal pigment epithelial cells (mRNA and protein) [23, 24]. Immunohistochemical studies supported the presence of CB2 receptors in retinal tissue of rats and mice [25, 26]. Specifically, the CB2 receptor was localized in the retinal pigment epithelium, inner photoreceptor segments, horizontal and amacrine cells, ganglion cell layer (GCL), and inner plexiform layer (IPL) of the rat retina and in all five neuronal types of the mouse retina. The presence of CB2 receptors in Müller cells of monkeys has also been reported [27]. However, different groups, employing radioligand binding studies in rat retinal membranes, suggested the absence or low undetectable levels of CB2 receptor in rat retina [28, 29]. Thus, further studies are needed in order to elucidate the presence and functional role of CB2 receptors in the rodent retina.

Two other receptor systems were shown to be activated by cannabinoids, namely, the transient receptor potential cation channel subfamily V member 1 (TRPV1 channel) and the orphan receptor GPR55 [30–34]. Immunohistochemical studies have shown the expression of TRPV1 channel in IPL, INL (inner nuclear layer), and GCL of adult rat retina, while GPR55 has been detected in rod photoreceptors of adult vervet monkey retina [33, 34]. The colocalization of GPR55 only with the inner segments of rod photoreceptors, with less immunoreactivity observed in peripheral retina regions, suggested that this asymmetric distribution of GPR55 in the monkey retina may underlie its function in phototransduction [34].

Both CB1 and CB2 cannabinoid receptors belong to the GPCR family [11–14]. The most well-known signal transduction response for both CB1 and CB2 receptors is the inhibition of adenylyl cyclase through interaction with Gi/o proteins [12, 35]. Activation of CB1 receptors affects G-protein-coupled inwardly rectifying potassium channels (GIRKs) [36, 37]. CB1 receptors interact with a variety of ion channels including Ca^2+^ and K^+^ channels [37–39].

### 2.2. Endocannabinoids in the Retina: Synthesis and Metabolism

AEA and 2-AG, as well as the enzymes responsible for their synthesis (N-acyl phosphatidylethanolamine phospholipase, D-NAPE-PLD, diacylglycerol lipase, DAGL) and degradation [fatty acid amide hydrolase (FAAH), monoacylglycerol lipase (MGL)], have been found in the retina of rodents and other mammals [8, 9, 22]. Immunohistochemical localization of DAGL has been reported in retinal tissue, especially in the postsynaptic terminals of cone bipolar cells. Yet despite the reported presence of NAPE-PLD in rat retina little is known about its specific localization in rat retinal neurons [40]. FAAH, the metabolic enzyme of AEA [41], has been shown to be localized in photoreceptor inner segments, horizontal, dopaminergic, and cholinergic amacrine and ganglion cells, as well as Müller cells, in the rodent retina [21, 22]. MGL, the metabolic enzyme of 2-AG [42], has also been reported in retinal tissue and specifically in the IPL and OPL (outer plexiform layer) [22].

### 2.3. Function of Cannabinoid CB1 Receptors in the Retina

As mentioned above, CB1 receptors can interact with a variety of ion channels [37–39]. In the retina CB1 receptor activation results in pertussis toxin sensitive actions (Gi/o-linked CB1 receptor), such as the modulation of ion channel function [43]. This cannabinoid function may influence retinal circuitry, neurotransmitter release, and neuroprotection. In retina, cannabinoids have been reported to modulate Ca^2+^ and/or K^+^ channels in bipolar cells and photoreceptors [10, 44–46]. The presence of CB1 receptors in rod bipolar cells and the CB1-dependent reduction in the amplitude of voltage-gated L-type calcium channel currents present in these retinal neurons suggest that endocannabinoids and synthetic cannabinoids may play an important role in retinal circuitry and in scotopic vision [21].

Glutamate is the major neurotransmitter in the retina released from photoreceptors, rod bipolar and ganglion cells. However, a plethora of other neurotransmitters is found in amacrine and horizontal cells. The presence of the CB1 receptor in all retinal neurons suggests that endocannabinoids and synthetic cannabinoids play a neuromodulatory role in retinal circuitry by influencing the release of other neurotransmitters. CB1 receptor agonists inhibited K^+^ induced [^3^H]-D-aspartate release from bovine retina, and Ca^2+^ evoked [^3^H]-noradrenaline and [^3^H]-dopamine release in guinea pig retina [47–49]. CB1 regulation of GABA release was reported by spontaneous mini frequencies involving GABA-A receptor-mediated inward currents, in cultured chick embryonic amacrine cells [50]. The authors of this study concluded that the “regulation of spontaneous transmitter release by endocannabinoids might be important in network maintenance in amacrine cells and other inhibitory interneurons.”

**Figure 1:**
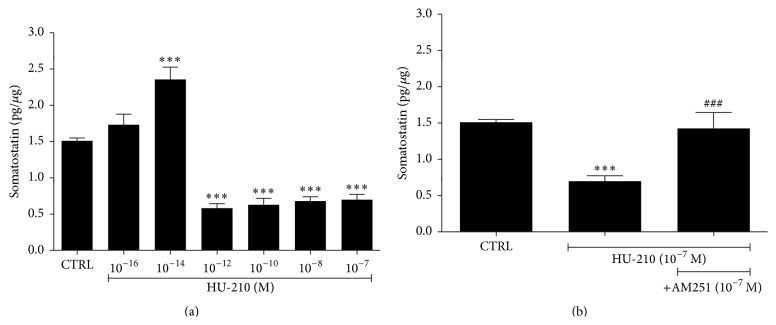
Effect of HU-210 on somatostatin's release in rat retina. (a) HU-210 at the low dose of 10^−16^ M had no effect on the release of somatostatin (1.7 ± 0.15 pg/*μ*g, *n* = 4) compared to the control tissue (CTRL, 1.5 ± 0.04 pg/*μ*g, *n* = 12). HU-210 at 10^−14^ M increased the release of somatostatin in the retina (2.4 ± 0.17 pg/*μ*g, *n* = 5, ^*∗∗∗*^ *p* < 0.001 compared to CTRL), whereas higher concentrations caused a statistically significant decrease in somatostatin's release (10^−12^ M, 0.6 ± 0.06 pg/*μ*g, *n* = 5, ^*∗∗∗*^ *p* < 0.001 compared to CTRL; 10^−10^ M, 0.63 ± 0.09 pg/*μ*g, *n* = 5, ^*∗∗∗*^ *p* < 0.001 compared to CTRL; 10^−8^ M, 0.68 ± 0.06 pg/*μ*g, *n* = 6, ^*∗∗∗*^ *p* < 0.001 compared to CTRL; 10^−7^ M, 0.7 ± 0.07 pg/*μ*g, *n* = 6, ^*∗∗∗*^ *p* < 0.001 compared to CTRL). Dunnett's Multiple Comparison Test was used for the statistical analysis of the data. All values represent the mean ± SEM. (b) Effect of the CB1 preferred antagonist AM251 in the actions of HU-210 (10^−7^ M) on somatostatin's release. AM251 (10^−7^ M) reversed the attenuation of somatostatin release by HU-210 (10^−7^ M) (1.42 ± 0.22 pg/*μ*g, ^###^ *p* < 0.001 compared to HU-210, *n* = 5). One-way ANOVA with Tukey's post hoc analysis test was used for the statistical analysis of the data. All values represent the mean ± SEM.

In rat retinal explants HU-210 attenuated the release of the inhibitory neuropeptide somatostatin in a dose-dependent bimodal manner via the activation of the CB1 receptor (Figure 1). Bimodal modulation was also reported in a study that showed that WIN55212-2 affected voltage-dependent currents of retinal cones in a biphasic manner [45].

The GPR55 receptor and the TRPV1 channel are also activated by cannabinoids, such as the endogenous cannabinoid AEA, the synthetic cannabinoid CP55940, and the nonpsychotropic cannabinoid CBD [30–34]. Recently, the presence of GPR55 receptor protein in rod photoreceptor cells of the vervet monkey was reported and the authors suggested that GPR55 activation in these cells may play a role in scotopic vision [34]. However, more studies are needed in order to evaluate the role of GPR55 receptor in retinal circuitry. The TRPV1 channel has been recently suggested to be involved in RGC function and survival in the retina [51]. Further studies regarding the neuroprotective actions of the TRPV1 channel in retina, upon its activation by the cannabinoids, are presented in Section 3.2.

## 3. Cannabinoids as Therapeutics in Retinal Disease

### 3.1. Ischemia-Induced Retinal Diseases

Glaucoma is the second cause of blindness worldwide and is characterized by elevation of IOP [52]. The main treatment of the disease to date involves the administration of drugs that reduce IOP by affecting either aqueous humour synthesis or outflow (cholinomimetics or cholinesterase inhibitors, alpha adrenergic receptor agonists, beta adrenergic receptor blockers, and others). The CB1 receptor was found to be localized in the anterior segment of the human and rat eye, including the ciliary body, epithelium, and the trabecular meshwork [53, 54]. These ocular tissues regulate aqueous humour inflow and outflow pathways and IOP. McIntosh et al. [55] reported that endogenous CB1 receptors couple with both Gq/11 and Gi/o, present in the trabecular meshwork, and elicit different responses according to the cannabinoid agonist employed in the study. Activation of CB1 receptors by WIN55,212-2, but not by CP55,940 or methanandamide, led to CB1 receptor coupling with Gq/11 and subsequent activation of PLC-dependent increase of intracellular Ca^2+^ levels. This study suggested that the differential effects of cannabinoid agonists in human trabecular meshwork cells may be used to identify cannabinoids that affect aqueous outflow and IOP. These pharmacological actions of cannabinoids render them useful therapeutic targets in lowering IOP in glaucoma patients.

However, the reduction of IOP is not sufficient to prevent visual field loss. The pathophysiology of glaucoma is multifactorial and recently more and more studies define glaucoma as a neurodegenerative disease, characterized by ischemia-induced excitotoxicity and loss of RGCs [56–59]. Elevated glutamate levels have been detected in the vitreous of humans and monkeys with glaucoma [60] and glutamate-induced excitotoxicity has been suggested to play a fundamental role in the RGCs loss observed in experimental glaucoma [61].

Diabetic retinopathy is another major ocular disease. While being defined by its microvascular characteristics (neovascularization), more recent reports suggest that it possesses neurodegenerative and inflammatory components [62]. In fact, it has been suggested that retinal function is compromised before the appearance of neovascularization in diabetic patients [63–65]. Oxidative stress has also been documented in both animals and diabetic patients and has been correlated with neuronal loss [66–68].

Many strategies have been used to develop therapeutic agents for the successful treatment of ischemia-induced retinopathies and the prevention of blindness [69]. The proneovascular agent vascular endothelium growth factor (VEGF) is considered to be the factor most responsible for the development of new vessels in diseases, such as proliferative DR. The discovery of anti-VEGF therapy has given new hope to patients, yet this treatment targets only the neovascular component of ischemia-induced retinopathies and has serious ocular adverse effects [70–72]. However, there are no therapeutics available to date to treat the neurodegenerative component of diabetic retinopathy or glaucoma (for a review, see [73]). In order to preserve vision, all three components of ischemia-induced retinopathies, neovascularization, neurodegeneration, and inflammation, must be addressed.

### 3.2. Cannabinoids as Neuroprotectants in Retinal Disease Models

Different animal disease models have been employed to examine the neuroprotective effects of putative therapeutics to treat neurodegenerative retinal diseases. These include* ex vivo* and* in vivo* models of ischemia and excitotoxicity, such as the* ex vivo* chemical ischemia model, the* ex vivo* and* in vivo* NMDA (N-methyl-D-aspartate) or AMPA (*α*-amino-3-hydroxy-5-methyl-4-isoxazolepropionic acid) models of excitotoxicity, and the* in vivo* animal models of IOP-reperfusion (glaucoma), and the streptozotocin (STZ) model of diabetic retinopathy [73].

**Figure 2:**
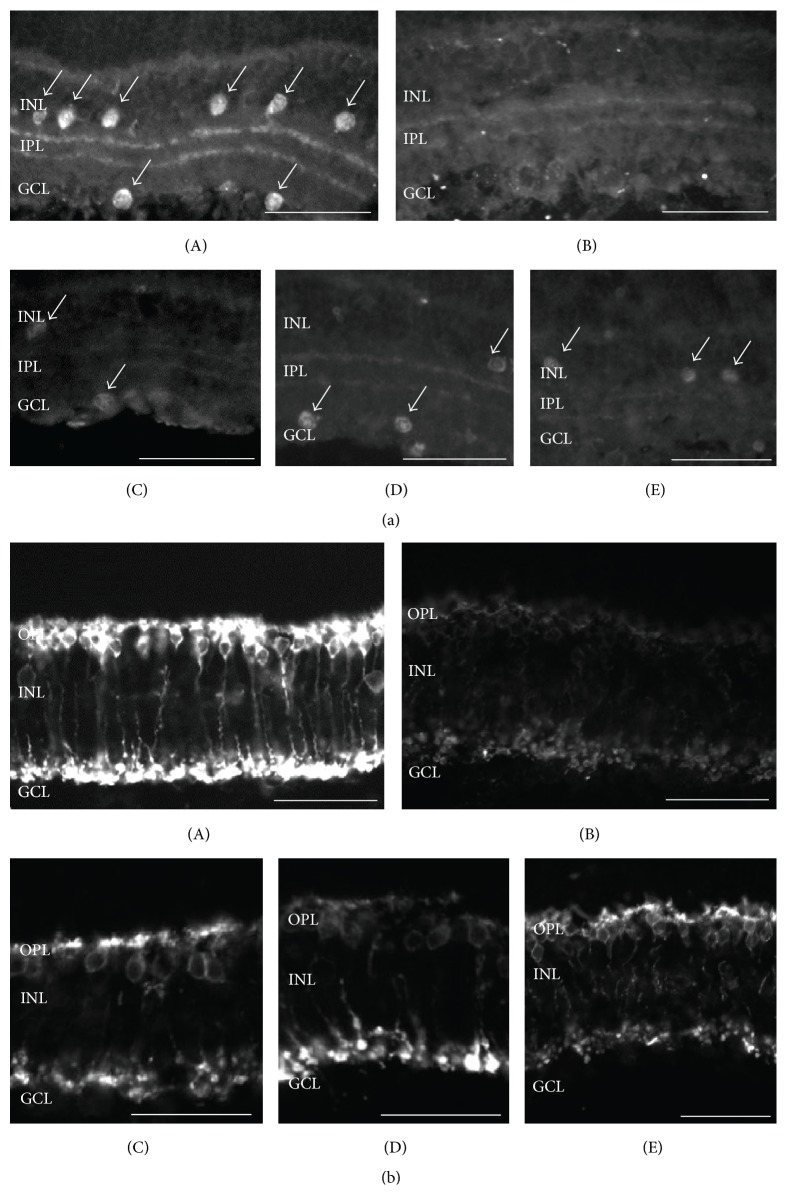
Effect of the synthetic CB1/CB2 cannabinoid HU-210 on ChAT and PKC immunoreactivity. (a) ChAT immunoreactivity. ChAT immunoreactivity in control tissue ((A) *n* = 12) is localized in cholinergic amacrine cell somata in the INL and GCL, as well as in their processes in the IPL. (B) Chemical ischemia mixture (*n* = 12) caused a reduction of ChAT immunoreactivity as revealed by loss of cholinergic cell somata and less intense signal in cell processes. HU-210 afforded neuroprotection at the concentrations of 10^−6^ M ((C) *n* = 5), 10^−5^ M ((D) *n* = 5), and 10^−4^ M ((E) *n* = 5). Arrows depict ChAT-immunoreactive amacrine cells. (b) PKC immunoreactivity. PKC immunoreactivity in control tissue ((A) *n* = 3) is localized in rod bipolar cells in the OPL and in their axons extending toward the IPL. Reduced immunoreactivity is observed in the presence of the chemical ischemia mixture ((B) *n* = 3). HU-210 afforded neuroprotection at all of concentrations used (10^−6^ M ((C) *n* = 3), 10^−5^ M ((D) *n* = 3), and 10^−4^ M ((E) *n* = 3)). Scale bar: 50 *μ*m. OPL: outer plexiform layer; INL: inner nuclear layer; IPL: inner plexiform layer; GCL: ganglion cell layer.

**Figure 3:**
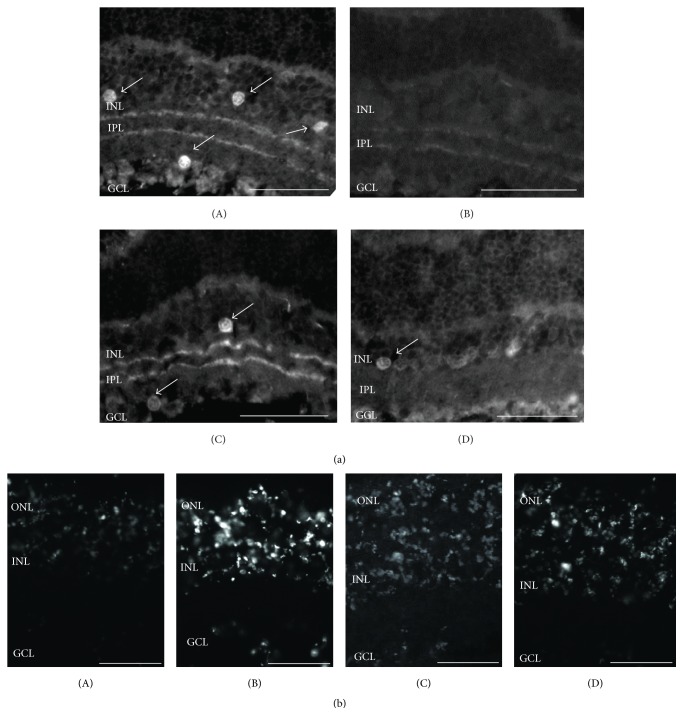
Involvement of the CB1 receptor on the neuroprotective actions of HU-210. (a) ChAT immunoreactivity. The selective inverse agonist AM251 attenuated the neuroprotective actions of HU-210 on cholinergic amacrine cells ((A) control, *n* = 12; (B) chemical ischemia mixture, *n* = 12; (C) HU-210 (10^−5^ M), *n* = 3; (D) HU-210 (10^−5^ M) + AM251 (10^−5^ M)). Arrows depict ChAT-immunoreactive amacrine cells. Scale bar: 50 *μ*m. (b) Effect of cannabinoids on retinal cell death. TUNEL staining was prominent in the chemical ischemia incubated tissues ((B) *n* = 2) while less staining was observed in the control tissues ((A) *n* = 2). TUNEL staining substantiates the neuroprotective effects of HU-210 ((C) 10^−5^ M, *n* = 2) and the reduced neuroprotection (increased TUNEL staining) in the presence of AM251 ((D) 10^−5^ M, *n* = 3). ONL: outer nuclear layer; INL: inner nuclear layer; IPL: inner plexiform layer; GCL: ganglion cell layer.

The* ex vivo* model of chemical ischemia involves the blockade of oxidative phosphorylation and glycolysis and is believed to be useful for the understanding of the early events underlying the pathophysiology of ischemia [74]. It was previously reported that chemical ischemia influences the viability of a variety of amacrine and rod bipolar cells, but not RGC or photoreceptor viability [75]. HU210 afforded neuroprotection to cholinergic amacrine and rod bipolar cells via activation of the CB1 receptor (Figures 2 and 3(a)). These results were also substantiated by TUNEL staining (Figure 3(b)).

The underlying cause of ischemia-induced cell death is excitotoxicity. Ischemic insults lead to activation of voltage gated calcium channels and increase in glutamate levels and activation of ionotropic glutamate (NMDA and AMPA) receptors leading to an excess of intracellular calcium ions [60]. NMDA has been the excitatory amino acid of choice as a model of ischemia-induced cell death in brain and retina. However, the AMPA excitotoxicity model has also been employed. Δ^9^-Tetrahydroxycannabinol (Δ^9^-THC; partly via a CB1 mechanism) and the non-CB1 agonist CBD were shown to protect the retina from NMDA excitotoxicity [76]. Both cannabinoids afforded neuroprotection to retinal neurons located in the INL and GCL via attenuation of lipid peroxidation and/or nitrotyrosine formation [76]. Intravitreal administration of AMPA in rat retina was shown to affect the viability of horizontal and bNOS, ChAT, and calbindin-expressing amacrine cells, but not photoreceptors, bipolar or ganglion cells [77]. This model has been employed to investigate the neuroprotective properties of new pharmacological agents in the early events of retinal ischemia* in vivo* [77–79]. In this paradigm of excitotoxicity, AEA and the synthetic cannabinoids HU-210 and MethAEA (nonhydrolysable analogue of AEA), intravitreally coinjected with AMPA, provided neuroprotection to bNOS and ChAT expressing amacrine cells [29]. The CB2 preferring agonist JWH015 did not display any neuroprotection. The selective inverse agonist AM251 reversed the neuroprotective actions of these agents. This study suggested that the CB1 receptor is responsible for the neuroprotective actions of the cannabinoids. In addition, AEA afforded neuroprotection to horizontal and GABA amacrine calbindin-immunoreactive cells [29].

The involvement of CB1 receptor in the neuroprotective actions of the endocannabinoid 2-AG has also been investigated in the AMPA excitotoxicity model. 2-AG was intravitreally coinjected with AMPA in wild-type, CB1 and CB2 C57BL/6 knockout mice. 2-AG reversed the AMPA induced reduction of bNOS expressing amacrine cells, in both wild-type and CB2^−/−^ mice. However, 2-AG did afford neuroprotection in the CB1 knockout retinas. These results substantiated the involvement of the CB1 receptor in the neuroprotective actions of 2-AG against AMPA excitotoxicity in mouse retina [80].

Cannabinoids are known to activate prosurvival downstream signaling pathways in different paradigms which involve, among others, the prosurvival PI3K/Akt and the MEK/ERK1/2 signaling pathways [81–85]. In rat retina, the PI3K/Akt signaling pathway was shown to be involved in the neuroprotective actions of HU-210 and AEA in the AMPA excitotoxicity model, whereas the MEK/ERK1/2 signaling pathway seemed to be involved in the neuroprotective actions of AEA, but not HU-210 [29]. Molina-Holgado et al. [86] also presented similar results reporting that HU-210 acted in a neuroprotective manner against AMPA excitotoxicity in primary cortical neuronal cultures by leading to the phosphorylation of Akt, but not ERK1/2 kinases. Moreover, MethAEA has also been shown to induce ERK1/2 phosphorylation in the hippocampus via a CB1-dependent mechanism [83].

In the* in vivo* IOP-reperfusion model of glaucoma, retinal ischemia-reperfusion led to an attenuation of AEA levels and this was shown to be due to the enhanced activity of its metabolic enzyme FAAH [28]. In this paradigm, a downregulation of CB1 receptors and TRPV1 channels was also reported. Administration of the FAAH inhibitor URB597 diminished the retinal damage, while intravitreal injection of MethAEA rescued RGCs via activation of CB1 receptors and TRPV1 channels [28]. This study supports that the endocannabinoid system may be involved in the loss of RGCs and that cannabinergic agents (CB1 or TRPV1 agonists and inhibitors of endocannabinoid metabolic enzymes) may be important therapeutics in glaucoma.

In the STZ animal model of diabetic retinopathy, CBD treatment provided neuroprotection, blood brain barrier preservation, and anti-inflammatory actions [87]. Thus, CBD prevented the neurodegenerative and neuroinflammatory components of diabetic retinopathy. Specifically, administration of CBD led to attenuation of proinflammatory cytokines, tumor necrosis factor (TNF*α*) and VEGF, reduction of oxidative and nitrative stress, and neuroprotection to inner retinal neurons [87].

Further support for the neuroprotection afforded by the synthetic cannabinoid HU210 to retinal neurons was shown in a study by Lax et al. [88]. These investigators performed immunohistochemical and electrophysiological studies and showed that HU210 afforded neuroprotection to photoreceptor cells in a model of retinitis pigmentosa [88]. Intraperitoneal administration of HU-210 improved the disrupted connectivity of photoreceptor cells with horizontal and bipolar cells and preserved the morphology and function of photoreceptors.

**Figure 4:**
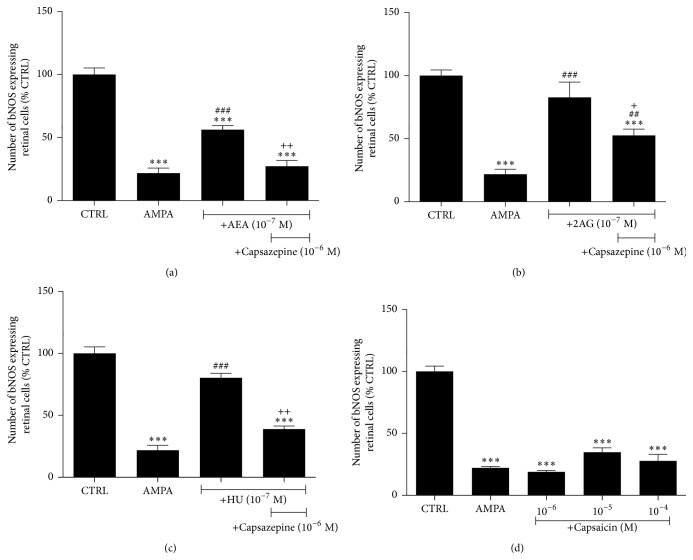
Involvement of the TRPV1 channel on the neuroprotective actions of endogenous and synthetic cannabinoids on bNOS expressing amacrine cells. (a) Intravitreal coinjection of the TRPV1 antagonist capsazepine (10^−6^ M) with AMPA (42 nmol/eye) + AEA (10^−7^ M, *n* = 6) or 2-AG (b) (10^−7^ M, *n* = 3) or HU-210 (c) (10^−7^ M, *n* = 3) attenuated the neuroprotective effects of the cannabinoid ligands (^*∗∗∗*^ *p* < 0.001 compared to CTRL, ^##^ *p* < 0.01, ^###^ *p* < 0.001 compared to AMPA, and ^+^ *p* < 0.05, ^++^ *p* < 0.01 compared to AEA (10^−7^ M) or 2-AG (10^−7^ M) or HU-210 (10^−7^ M), resp.). (d) The TRPV1 channel agonist capsaicin (10^−6^–10^−4^ M, *n* = 3) coinjected with AMPA had no neuroprotective effect at any of the doses tested (^*∗∗∗*^ *p* < 0.001, compared to CTRL). One-way ANOVA with Tukey's post hoc analysis was used for the statistical analysis of all data presented in this figure.

Cannabinoids also bind to and activate TRPV1 channels. Previous studies suggested that the TRPV1 ligand capsaicin induces degeneration of RGCs in preweanling rats [89] and apoptosis in isolated RGCs via a Ca^2+^-dependent mechanism [90]. However, capsaicin has been shown to protect RGCs in an* in vivo* model of NMDA retinal excitotoxicity in rats, via TRPV1 channel activation [91]. Intravitreal administration of MethAEA via activation of the TRPV1 channel rescued RGCs from ischemia-reperfusion insults [28]. The involvement of the TRPV1 channel in the neuroprotective properties of AEA, 2-AG, and HU-210 was also examined in the AMPA excitotoxicity model. The TRPV1 antagonist capsazepine intravitreally administered with the above agents attenuated their neuroprotective actions on the bNOS-immunoreactive amacrine cells, suggesting a neuroprotective role for the TRPV1 channel in this paradigm (Figures 4(a)–4(c)). However, functional studies employing the TRPV1 agonist capsaicin did not show any neuroprotective effects at any of the doses used (Figure 4(d)). These results suggested that the TRPV1 channel is not localized in bNOS amacrine cells and that capsazepine's actions are independent of the TRPV1 channel.

The results presented in this part of the review provide strong evidence for the neuroprotective role of the endocannabinoids and the synthetic cannabinoids in different models of retinal disease via the activation primarily of the CB1 receptor and its signal transduction mechanisms. In Section 3.3 the interaction of the endocannabinoid system with other neuromodulatory systems in the retina will be presented in order to assess the influence of this interaction on the cannabinoid induced neuroprotection.

### 3.3. Neuromodulatory Role of Cannabinoids and Neuroprotection

The CB1 receptor has been localized in photoreceptors, rod bipolar, a variety of amacrine, horizontal, and ganglion cells in retinas of many species. This was assessed by immunohistochemical studies [10, 21] and also confirmed from the functional neuroprotective roles of the endogenous and synthetic cannabinoids in the different models of retinopathy presented in this review. The localization of the CB1 receptor in each retinal neuron provided in many cases information on the mechanism involved in the neuroprotection of the agents employed.

The presence of the CB1 receptor in rod bipolar cells suggests that its activation may influence glutamate neurotransmission. WIN55,212-2 was shown to inhibit L-type calcium currents in salamander bipolar cells [10]. These channels regulate glutamate release and their inhibition will modulate in a negative manner glutamatergic transmission between the bipolar, amacrine, and ganglion cells. While release studies have not been performed on isolated rod bipolar cells, CB1 agonists were shown to inhibit K^+^ and ischemia-induced [^3^H]-D-aspartate release from isolated bovine retinas, as mentioned above [47]. A decrease in glutamate and intracellular calcium ion levels will lead to neuroprotection, since the underlying cause of excitotoxicity induced cell death is the rise in intracellular calcium levels. A possible mechanism via which HU-210 afforded neuroprotection to rod bipolar cells in the model of chemical ischemia (Figure 2) may involve the direct activation of CB1 receptors and the subsequent inhibition of calcium and glutamate levels.

A similar conjecture cannot be made as to the mechanisms via which HU-210 provided neuroprotection to ChAT-immunoreactive amacrine cells. While the CB1 receptor has been localized in subpopulations of amacrine cells, its presence in ChAT expressing neurons, to our knowledge, has not been reported to date [10, 21]. One cannot exclude the presence of the CB1 receptor in cholinergic neurons, yet neuroprotection may also be afforded by cannabinoids via indirect mechanisms orchestrated by the modulatory actions of cannabinoids and other neurotransmitters in the retina, as will be presented in the following.

Inhibitory neurotransmitters, such as GABA, could counteract the toxic influence of glutamate on retinal neurons during retinal ischemia and would be expected to provide neuroprotection. GABA was also suggested as a neuroprotective agent in brain acute ischemic stroke [92]. A reduction of the excitatory input or an increase of the inhibitory input in the retina can afford neuroprotection.

Dopamine (DA) is a major neuromodulator in the retina that affects retinal circuitry by activating two major families of dopamine receptors, D_1_ and D_2_ [93, 94]. CB1 and the dopamine inhibitory D_2_ receptors are found in cones, whereas CB1 and the dopamine stimulatory D_1_ receptors are found in ON bipolar cells. A reciprocal inhibition of voltage-gated potassium currents (*I*K_(*V*)_) by activation of cannabinoid CB1 and dopamine D_1_ receptors in ON bipolar cells of goldfish retina was reported [46]. These findings support an antagonistic interaction between the cannabinoid and dopamine signaling that may influence transmitter release. In addition, it was proposed that endocannabinoids function as a scotopic signal, interacting with dopamine to set retinal sensitivity.

DA also regulates the release of other retinal neuromodulators, such as NO (nitric oxide) and the neuropeptide somatostatin. It enhances NO release, while a reciprocal effect was also observed, namely, NO attenuating DA release [95–97]. NO, cGMP, and the NO donor SIN-1 stimulated the release of GABA in the retina [98]. Somatostatin was also shown to influence the release of both dopamine and NO in the retina [99, 100]. DA, via D_1_ receptor activation, and NO, via guanylyl cyclase and cGMP, influence somatostatin levels in the retina. These results suggested that the triad of neuromodulators, somatostatin-dopamine-NO, have reciprocal interactions via which they regulate retinal circuitry [101]. CB1 agonists were also shown to inhibit the release of retinal [^3^H] dopamine [48]. HU-210 regulated somatostatin release in a bimodal manner (Figure 1(a)), in agreement with what was reported for cannabinoid effects on GABA release in the globus pallidus [102]. Therefore, it appears that the interactions amongst transmitter and neuromodulator systems in the retina not only regulate retinal circuitry, retinal sensitivity, and light adaptation but may also provide the neuroprotective mechanisms involved in reducing ischemia-dependent toxicity. It is possible that cannabinoid interactions with other retinal modulatory systems may lead to the indirect neuroprotection of retinal neurons.

However, other mechanisms may also be involved. HU-210 was shown to provide neuroprotection and counteract the oxidative stress and cellular injuries observed in diabetic encephalopathy, a CNS neuropathy and one of the most common complications of diabetes, through a cannabinoid receptor-independent mechanism [94].

## 4. Concluding Remarks

The aim of this review was to summarize the knowledge acquired to date on the function of endogenous and synthetic cannabinoids in the retina, the receptors they activate to mediate their actions, and the neuroprotective role of cannabinoids in retinal models of disease. The studies presented in this review support the involvement of the endocannabinoids, AEA and 2-AG, and synthetic cannabinoids in the neuroprotection of the early and final events underlying the pathophysiology of retinal ischemia. It appears that these agents have the pharmacological profile to target the neurodegenerative/proapoptotic components of retinal disease.

The available treatments for neurodegenerative retinal diseases have limitations and further research is needed in order to provide efficacious therapies. Cannabinergic agents, CB1 or TRPV1 agonists and inhibitors of endocannabinoid metabolic enzymes, may prove to be efficacious therapeutics in the treatment of glaucoma and diabetic retinopathy.

In order to provide “LEAD” cannabinergic agents, further studies are essential (a) to study the pharmacokinetic properties of cannabinoids that would suggest their use as effective topical retinal therapies and (b) to ascertain their pharmacological efficacy in chronic use. These studies will assess whether cannabinergic agents can play an important role alone or in a multidrug treatment to provide efficacious therapy and improve the eye sight of millions of people worldwide who are afflicted with neurodegenerative retinal disease.

## Abbreviations

2-AG: 2-Arachidonoylglycerol
ACEA: Arachidonyl-2′-chloroethylamide
AEA: Arachidonoyl ethanolamide
AMPA: *α*-Amino-3-hydroxy-5-methyl-4 isoxazolepropionic acid
bNOS: Brain nitric oxide synthase
CB1: Cannabinoid receptor 1
CB2: Cannabinoid receptor 2
CBD: Cannabidiol
cGMP: Cyclic guanosine monophosphate
ChAT: Choline acetyl transferase
CNS: Central nervous system
DAGL: Diacylglycerol lipase
ERK1/2: Extracellular signal-regulated kinases 1/2
FAAH: Fatty acid amide hydrolase
GABA: Gamma-aminobutyric acid
GCL: Ganglion cell layer
INL: Inner nuclear layer
IOP: Intraocular pressure
IPL: Inner plexiform layer
MethAEA: Methanandamide
MGL: Monoacylglycerol lipase
NAPE-PLD: N-Acyl phosphatidylethanolamine phospholipase D
NMDA: N-Methyl-D-aspartate
Δ^9^-THC: Δ^9^-Tetrahydroxycannabinol
NO: Nitric oxide
ONL: Outer nuclear layer
OPL: Outer plexiform layer
PKC: Protein kinase C
RGC: Retinal ganglion cell
SAPK/JNK: Stress-activated protein kinase/c-Jun N-terminal
STZ: Streptozotocin
TH: Tyrosine hydroxylase
TRPV1: Transient receptor potential cation channel subfamily V member 1
TUNEL: Terminal deoxynucleotidyl transferase (TdT) dUTP nick-end labeling
VEGF: Vascular endothelial growth factor.
